# Comparison of Three Different Balanced Sedative-Anaesthetic Protocols in Captive Baboons *(Papio hamadryas)*

**DOI:** 10.3390/vetsci12090859

**Published:** 2025-09-04

**Authors:** Martina Amari, Federica Alessandra Brioschi, Petra Cagnardi, Giulia Sala, Francesco Ferrari, Michele Capasso, Luigi Elia, Elena Venturelli, Federica Di Cesare, Francesco Zinno, Giuliano Ravasio

**Affiliations:** 1 Department of Veterinary Medicine and Animal Sciences (DIVAS), University of Milan, Via dell’Università 6, 26900 Lodi, Italy; martina.amari@unimi.it (M.A.); petra.cagnardi@unimi.it (P.C.); francesco.ferrari@unimi.it (F.F.); luigi.elia@guest.unimi.it (L.E.); giuliano.ravasio@unimi.it (G.R.); 2 Department of Veterinary Sciences, University of Pisa, Viale delle Piagge 2, 56124 Pisa, Italy; giulia.sala@unipi.it; 3 Department of Veterinary Medicine and Animal Production, University of Naples Federico II, Via Federico Delpino 1, 80138 Napoli, Italy; capassovet@gmail.com; 4 Zoo Safari Ravenna, Via dei Tre Lati 2/X, 48125 Ravenna, Italy; franz.zinno@gmail.com; 5 Clinica Veterinaria di Russi, Via Faentina Nord 125, 48026 Russi, Italy; venturellielena017@gmail.com

**Keywords:** chemical restraint, blood gas analysis, dexmedetomidine, ketamine, non-human primates, methadone, midazolam, recovery quality, tiletamine-zolazepam

## Abstract

Baboons’ management requires chemical restraint. Three intramuscular sedative protocols in captive hamadryas baboons (*Papio hamadryas*) undergoing health-check and male vasectomy were compared. Animals were assigned to TZD_G (*n* = 17; tiletamine/zolazepam 3 mg/kg + dexmedetomidine 20 μg/kg), KDM_G (*n* = 23; ketamine 6 mg/kg + dexmedetomidine 30 μg/kg + methadone 0.2 mg/kg), or MDM_G (*n* = 9; midazolam 2 mg/kg + dexmedetomidine 60 μg/kg + methadone 0.2 mg/kg). Propofol was titrated intravenously for anaesthetic induction and maintenance. Sedation time and quality and cardiopulmonary parameters were recorded. Atipamezole (TZD_G 0.2 mg/kg, KDM_G 0.3 mg/kg, MDM_G 0.6 mg/kg) and flumazenil (MDM_G 0.02 mg/kg) were administered intramuscularly post-procedure. Recovery time and quality were recorded. Data were reported as median (interquartile range) or regression coefficient (B). Sedation was deepest in TZD_G (20, 20–20; KDM_G 20, 19–20; MDM_G 19, 15–20; *p* = 0.017). MDM_G had a significantly higher heart rate (B = 10.27, *p* = 0.001), respiratory rate (B = 9.09, *p* < 0.001), and lower end-tidal carbon dioxide (B = −3.00, *p* = 0.03) than TZD_G, while KDM_G had a lower respiratory rate than TZD_G (B = −3.67, *p* = 0.02) and a higher temperature (B = 1.66 *p* = 0.001). TZD_G showed the longest recovery (minutes: 19, 11.5–30; KDM_G: 6, 4–12; MDM_G: 4, 2.5–5; *p* < 0.001), while MDM_G the best recovery (0, 0–0; TZD_G: 9, 6–12; KDM_G: 0, 0–6; *p* < 0.001). TZD produced deepest sedation but bad recovery; KDM offered optimal sedation and recovery, and cardiopulmonary stability; MDM provided lighter sedation and excellent recovery.

## Simple Summary

Routine health checks and minor procedures in captive hamadryas baboons require safe and reliable sedation protocols that ensure animal health and staff safety. This study evaluated the clinical effects of three intramuscular drug combinations: (1) tiletamine-zolazepam with dexmedetomidine; (2) ketamine combined with dexmedetomidine and methadone; and (3) midazolam with methadone and dexmedetomidine. Propofol was administered for induction and maintenance of general anaesthesia. Intramuscular atipamezole (in all protocols) and flumazenil (in protocol 3) were given post-procedure to enhance recovery. All protocols provided adequate sedation and stable cardiopulmonary parameters. The first protocol produced the deepest sedation, but was associated with prolonged and variable recovery, making it particularly suitable for emergencies requiring rapid and effective immobilization. The second protocol provided optimal sedation, stable anaesthesia, acceptable gas exchange, and good recovery quality, emerging as the most balanced and effective option for general anaesthesia. The third protocol produced lighter sedation and the fastest and smoothest recoveries, making it ideal for minor/short procedures. These findings support a tailored approach to sedation in hamadryas baboons, allowing clinicians to select the most appropriate drug combination to ensure animal welfare, staff safety, and the potential promptness of returning individuals to their social group.

## 1. Introduction

Baboons (*Papio* spp.) are maintained in zoological institutions worldwide for a variety of purposes, including education, scientific research, and both in situ and ex situ conservation through managed breeding programs. Research involving these non-human primates (NHPs) has significantly contributed to the understanding of their behaviour, social organization, and biology [1]. However, routine management and veterinary procedures in captive NHPs often carry a substantial risk of injury to personnel, including scratches, bites, fractures, and exposure to potentially serious zoonotic infections such as *Mycobacterium tuberculosis* [2,3].

To minimize these risks and improve both safety and animal welfare, chemical restraint through sedation or anaesthesia is widely recommended. These pharmacological strategies are essential not only to facilitate sample collection and diagnostic or therapeutic procedures but also to reduce stress-related complications, such as capture myopathy associated with physical restraint [3,4]. Reflecting this, the Association of Primate Veterinarians has recently updated its guidelines to highlight the critical role of chemical restraint in the safe and ethical handling of NHPs [5]. Anaesthesia also plays a central role in population control strategies within zoological institutions. In highly prolific species such as baboons, it enables the implementation of contraceptive measures to prevent overpopulation and mitigate the risks of inbreeding within managed colonies [6].

Ketamine is one of the most widely used anaesthetic agents in NHPs, due to its rapid onset and cardiopulmonary stability [7]. However, when administered alone, ketamine provides limited muscle relaxation and is often associated with prolonged and poor-quality recoveries [7]. Therefore, it is commonly combined with other agents, such as benzodiazepines or α_2_-agonists. These balanced protocols enable dose reduction and improved anaesthetic depth and muscle relaxation, while minimizing adverse effects. In olive baboons, the addition of diazepam to ketamine resulted in enhanced muscle relaxation compared to ketamine alone [8]. Similarly, the ketamine-xylazine combination has been successfully employed in several *Papio* species, offering smoother induction, easier intubation, and longer anaesthetic duration [9,10]. Ketamine combined with medetomidine has also yielded favorable results in various NHP species, providing deeper anaesthesia with greater muscle relaxation, absent or minimal reflexes and spontaneous movements, without clinically relevant cardiorespiratory effects, compared to ketamine alone [11]. Similar results have been reported in *Papio hamadryas* when ketamine was used in combination with both medetomidine and midazolam [12]. Dexmedetomidine, a potent and highly selective α_2_-agonist [13,14], has been successfully used in macaques and tamarins [15,16], but, to the best of the authors’ knowledge, its use in baboons has not yet been reported.

Beyond ketamine-based regimens, other dissociative anaesthetic combinations such as tiletamine, in combination with zolazepam, have also been investigated in primates [17,18]. While these agents maintain acceptable cardiopulmonary function, a protocol combining tiletamine–zolazepam and medetomidine in *Papio hamadryas* was recently associated with delayed and low-quality recoveries [19]. By contrast, protocols excluding dissociative agents, such as α_2_-agonist and midazolam-based combinations, have been evaluated as alternatives for short procedures, offering rapid onset of sedation and rapid uneventful recovery in Japanese macaques [20] and howler monkeys [21]. Indeed, the ability to fully reverse anaesthetic agents is particularly advantageous in highly social species like baboons, where rapid post-anaesthetic reintegration into the social group is critical to maintain social cohesion and reduce stress [12].

The use of opioids in NHPs for anaesthesia and analgesia remains a subject of ongoing debate. While opioids are routinely employed for pain management and to reduce the requirement for inhalant anaesthetics, generally without causing significant cardiovascular effects [22], they may induce significant respiratory depression in primates [7]. Agents such as butorphanol and fentanyl have been associated with marked respiratory depression in NHPs, suggesting an increased species-specific sensitivity [21,23,24,25]. In contrast, milder respiratory effects have been reported following the administration of methadone or buprenorphine [26,27], making them potentially safer options in this context.

Based on current evidence, the aim of the present study was to evaluate and compare the sedative effects, cardiopulmonary stability, and recovery quality of three sedative–anaesthetic protocols in *Papio hamadryas*, undergoing routine health examinations, microchip implantation, and vasectomy in males. The first protocol includes tiletamine–zolazepam combined with dexmedetomidine, a regimen not yet described in this species but previously used with success in other primates. The second consists of ketamine combined with dexmedetomidine and methadone (KDM), reflecting the widespread use of ketamine–α_2_-agonist combinations and the relatively mild respiratory effects associated with methadone. The third protocol comprises midazolam, dexmedetomidine, and methadone, a fully reversible combination that may be particularly advantageous in socially complex species requiring prompt post-anaesthetic reintegration. The hypothesis was that all three sedative–anaesthetic protocols would provide effective sedation and stable cardiopulmonary parameters, without inducing adverse effects in *Papio hamadryas*. However, midazolam-dexmedetomidine-methadone (MDM) would result in lighter sedation, while tiletamine-zolazepam-dexmedetomidine (TZD) might have been associated with prolonged recoveries.

## 2. Materials and Methods

### 2.1. Animals

The present study complies with ethical standards and was approved by the Institutional Ethical Committee for Animal Care at the University of Milan (OPBA_74_27/08/2024). Private owner informed written consent was obtained.

Captive hamadryas baboons (*Papio hamadryas*) from the colony at Safari Ravenna Wildlife Park (Savio di Ravenna, Italy) were anesthetized for routine health examinations, microchipping, and vasectomy in males, as part of a population control program aimed at preventing further growth and reducing welfare concerns related to high animal density. The socially structured group was housed in a facility comprising an indoor recovery area and a spacious outdoor exhibit. The diet consisted of daily fresh fruits and vegetables, alfalfa hay, and a weekly portion of boiled chicken and rice. All apparently healthy individuals, defined by normal food and water intake, typical social behaviour, and regular motor activity during the 10 days preceding the procedure, were considered eligible. Infants, identified by their complete dependence on the mother and constant transport on the ventral or dorsal surface, as well as their lactating mothers, were excluded from all procedures for ethical and welfare reasons.

### 2.2. Study Design

Animals were fasted for 12 h before anaesthetic procedure, but water remained available. On the morning of the procedures, animals were kept in their indoor enclosure, where they had been confined the previous evening. To minimize stress, groups of three baboons were separated from the main group using a system of adjacent transfer cages by trained animal keepers, following conditioning procedures previously implemented; the interval between separation and anaesthetic administration was kept under 10 min. Eligible animals were randomly assigned (www.randomizer.org, accessed on 18 November 2023) to one of three treatment groups. Group TZD (TZD_G) received an intramuscular (IM) administration of tiletamine/zolazepam (total 3 mg/kg, comprising 1.5 mg/kg tiletamine HCl and 1.5 mg/kg zolazepam HCl) (Zoletil 100 mg/mL, containing 50 mg/mL of each component, Virbac S.r.l., Milan, Italy) and dexmedetomidine (20 µg/kg) (Dexdomitor 0.5 mg/mL; Vetoquinol Italia S.r.l., Forlì, Italy); baboons in group KDM (KDM_G) were administered IM ketamine (6 mg/kg) (Lobotor 100 mg/mL; ACME S.r.l., Florence, Italy), dexmedetomidine (30 µg/kg), and methadone(0.2 mg/kg) (Semfortan; Dechra Veterinary Products, Monza, Italy); group MDM (MDM_G) received IM midazolam (2 mg/kg) (Midazolam Hameln 5 mg/mL; Hameln pharma gmbh, Hameln, Germany), dexmedetomidine (60 µg/kg), and methadone (0.2 mg/kg). Drug doses were calculated based on body weight estimated by a veterinarian experienced in NHPs. Syringes containing the anaesthetic mixtures were prepared and labelled in a blinded manner to ensure allocation concealment. The IM injections were performed in the thigh muscle using inject high-performance Blowpipes B16 (TELEDART GMBH & Co. KG, Westheim, Germany) by three experienced veterinarians. All individuals within each trio were darted simultaneously to minimize stress. Anaesthetic procedures and monitoring were conducted by a separate team of three experienced anaesthetists blinded to group allocation. A supervising anaesthetist, who was aware of the assigned treatments, was present solely to ensure animal safety throughout the procedures. Following drug administration, each baboon was monitored for sedation. The time to seated position and to lateral recumbency from the drug administration were recorded. At 10 min post-injection, animals were approached only if they exhibited no movement in response to gentle tactile stimulation of the foot or hand. In cases of inadequate sedation, a supplemental intramuscular dart was administered using 50% of the original dose of each agent in the chosen protocol. Possible causes included underestimation of the animal’s body weight, suboptimal dart pressurization leading to partial or failed discharge, and incomplete drug delivery associated with premature needle dislodgement within the muscle. The total number of supplemental darts was recorded. Sedation quality was assessed using the scale described by Bertrand and colleagues (2016), assigning a score from 1 to 5 for each of the following parameters: movement, palpebral reflex, jaw tone, and withdrawal reflex [28]. A higher total score indicated a deeper level of sedation [28].

Each subject was weighed and placed on a warming pad covered with an absorbent blanket. Three anaesthesiologic workstations were prepared to allow simultaneous procedures: one for medical checkup and microchipping, one for ultrasonographic procedures and one for surgery; the animals were transported from one station to the next until all necessary treatments were completed. All animals underwent a complete physical examination to assess general health and assign an age class. Particularly, juveniles showed no signs/rudimental development of sexual characteristics, subadults exhibited partially developed sexual characteristics, and adults presented with fully developed sexual characteristics. An identification microchip was implanted subcutaneously (SC) at the base of the left side of the neck. A 20- or 22-gauge peripheral venous catheter (Jelco IV Catheter Radiopaque; Smiths Medical Italia S.r.l., Bologna, Italy) was inserted into the cephalic vein, and 3 mL of blood were collected for health screening, according to standard veterinary protocols for non-cooperative zoo primates. From the same venous sample, haematological, biochemical, and serum protein electrophoresis tests were conducted. The results of these analyses are the subject of a separate study [29]. Subsequently, general anaesthesia was induced by intravenous (IV) administration of propofol (Proposure, Merial Italia S.p.A., Milan, Italy). At the time of orotracheal intubation, patients in deep sedation were defined as those exhibiting a laryngeal reflex and therefore received propofol titrated to effect to achieve intubation, whereas patients “anesthetized” or under general anesthesia were defined as those without a laryngeal reflex and did not require propofol for intubation. Orotracheal intubation was performed after topical application of 1 mL of lidocaine (Lidocaina Cloridrato 2%; Salf S.p.A., Bergamo, Italy) to the arytenoid cartilages. This time was considered the T0. Anaesthetic depth throughout the procedure was assessed by evaluating the presence or absence of the palpebral reflex and jaw tone through passive mouth opening. In the event of spontaneous movement, preserved muscle tone, or persistence of palpebral reflex, the anaesthetic plan was deemed inadequate, and additional propofol (0.5 mg/kg IV) was administered. The total dose of propofol required for induction and maintenance was recorded. Lactated Ringer’s solution (Ringer Lattato, Fresenius Kabi, Mirandola, Italy) was infused IV at 2 mL/kg/h. A 22-gauge arterial catheter (Jelco IV Catheter Radiopaque; Smiths Medical Italia S.r.l., Bologna, Italy) was aseptically placed in the metatarsal artery and 15 min after the induction, an arterial blood gas analysis was performed (Epoc NXS Host; Epocal Inc.; Ottawa, ON, Canada). Analysis included arterial pH, partial pressure of arterial oxygen (PaO_2_) and arterial carbon dioxide (PaCO_2_), alveolar-arterial (A-a) gradient, a/A ratio (a/A%), bicarbonate (HCO^3−^) electrolytes (Na^+^, K^+^, Ca^++^, Cl^−^), base excess (BE), haematocrit (HCT), haemoglobin (Hgb), glucose, lactate, blood urea nitrogen (BUN), urea and creatinine. Heart rate (HR), invasive systolic, mean, and diastolic blood pressures (SAP, MAP, DAP, respectively), respiratory rate (RR), peripheral oxygen saturation (SpO_2_), end-tidal carbon dioxide concentration (ETCO_2_) and rectal body temperature (T) were continuously monitored throughout the anaesthesia and recorded every 10 min using three multiparameter anaesthesia monitor (Dräger Vista 120S, Dräger Italia Spa, Corsico, Italy). Ventilation support, via an anaesthetic circuit and 100% oxygen, was provided if respiratory depression (ETCO_2_ > 60 mmHg and PaO_2_ < 60 mmHg) or apnoea occurred [30,31]. In case of hypotension, defined as MAP lower than 60 mmHg, an IV crystalloid bolus (10 mL/kg lactated Ringer’s solution, over 10 min) was given. Side effects were also recorded.

Transthoracic echocardiography was performed in all subjects to screen for potential cardiac abnormalities. This procedure is especially relevant in light of the findings by Dick et al. (2014), who reported that cardiovascular diseases accounted for 12.2% of deaths (more than one in ten) in a series of 4350 captive baboon necropsies [32]. All males received prophylactic antibiotic therapy with IV cefazolin (20 mg/kg) (Cefazolina Teva, Teva Italia S.r.l., Milan, Italy) 15 min before skin incision. Animals were placed in dorsal recumbency, and the surgical area was clipped and aseptically prepared. Vasectomies were performed by the same experienced surgeon. Intraoperative nociception, defined as a ≥20% increase in HR, RR, or MAP relative to pre-incision values, was managed with IV fentanyl at a dose of 2 µg/kg (Fentadon, Dechra Veterinary Products, Turin, Italy) [19,33]. The number of fentanyl boluses and total surgical time were recorded. At the end of the procedure, meloxicam 0.2 mg/kg SC (Metacam, Boehringer Ingelheim, Milan, Italy) was administered for post-operative analgesia. Female subjects underwent transthoracic echocardiography and abdominal ultrasonography to detect potential pregnancy status or uterine organ abnormalities.

At the end of each procedure, baboons were extubated, arterial and venous access were removed, and they were placed in warmed recovery cages under infrared lamps in a quiet, isolated environment. All subjects received an IM dose of atipamezole (Antisedan 5 mg/mL; Vetoquinol Italia S.r.l., Forlì, Italy): 0.2 mg/kg (TZD_G), 0.3 mg/kg (KDM_G), or 0.6 mg/kg (MDM_G). Animals in the MDM_G also received flumazenil (0.02 mg/kg IM) (Flumazenil Kabi 0.1 mg/mL; Fresenius Kabi Italia S.r.l., Mirandola, Italy), combined in the same syringe. Antagonists were prepared and administered by the supervising anaesthetist to maintain treatment blinding. Anaesthesia duration (from the dart injection until the antagonists’ administration) and recovery time, namely the time from the injections of the antagonists to the standing position, were recorded. Recovery quality was assessed using the scale developed by Bertrand et al. (2016), which ranged from 0 to 15 points [28]. The scale scored the eye position, signs of nausea or vomiting, body posture, ataxia, and level of environmental awareness, with higher scores indicating poorer recovery quality [28]. Once animals had fully recovered and were deemed suitable for reintegration by a veterinarian experienced in NHPs, they were returned to their social group.

### 2.3. Statistical Analysis

Sample size was calculated using G*Power (v. 3.1, Franz Faul, Edgar Erdfelder, Albert-Georg Lang, and Axel Buchner, 2006, 2009, Düsseldorf, Germany), applying a repeated measure analysis of variance (ANOVA) with both within-and between-group comparisons across three groups (TZD_G, KDM_G and MDM_G) and seven time-points (T0, T10, T20, T30, T40, T50, T60). Sample size was calculated using heart rate as the primary variable, assuming an effect size of 0.2 [34], a significance level (α) of 0.05 (two-tailed), and a power of 95%. The power analysis indicated that a minimum total sample size of 48 baboons was required.

All statistical analyses were performed using IBM SPSS Statistics (version 29.0, IBM Corp., Armonk, NY, USA). Data were first analysed descriptively. Continuous variables were reported as median and interquartile range (25th–75th percentile) because the data were not normally distributed (Shapiro–Wilk test), while categorical variables were expressed as absolute frequencies and percentages. To compare demographic (age, gender, body weight) and clinical variables among the three treatment groups (TZD, KDM, MDM), the Kruskal–Wallis test was used for continuous variables, and the Chi-square test was applied to categorical variables. When significant differences were found, Bonferroni-adjusted post hoc comparisons were conducted. To assess within-group time-dependent changes in repeated physiological parameters (HR, RR, SAP, MAP, DAP, T, ETCO_2_ and SpO_2_), a one-way repeated-measures ANOVA was performed separately for each group. When the main effect of time was significant, post hoc pairwise comparisons with Bonferroni correction were carried out. To evaluate between-group effects across time, a Generalized Estimating Equations (GEE) model was applied, assuming a Poisson distribution. This model was used to assess whether the treatment group (fixed effect) significantly influenced the overall trend of physiological variables over time (dependent variable). GEE results are presented as regression coefficients (B), which represent the estimated mean difference between groups, along with associated *p*-values. For all analyses, a *p*-value < 0.05 was considered statistically significant.

## 3. Results

Out of a total of 55 hamadryas baboons, 49 hamadryas baboons were included in the present study (TZD_G *n* = 17; KDM_G *n* = 23; MDM_G *n* = 9), while three infants and their mothers (total *n* = 6) were excluded for ethical and welfare reasons. Results regarding the demographic data of the animals and the actual drug dosages administered, distributed across the three treatment groups, are presented in Table 1. Based on age classification, 14 baboons (28.6%) were categorized as juveniles, 5 (10.2%) as sub-adults, and 30 (61.2%) as adults. No statistical differences in age distribution were detected between groups (*p* = 0.74). Of the 49 baboons included, 29 (59.2%) were males and 20 (40.8%) were females. A significant difference in gender distribution was observed between groups (*p* = 0.03). Estimated and actual body weight differed significantly between groups (*p* = 0.007, both), with post hoc analysis showing a significant difference in both estimated and actual weights between KDM_G and MDM_G (*p* = 0.006, both).

During sedation, supplemental darts were required in 0/17 (0%) cases of TZD_G, 5/23 (21.7%) cases of KDM_G, and 3/9 (33.3%) cases of MDM_G. Comparisons revealed a significant difference between TZD_G and KDM_G (*p* = 0.04) and between TZD_G and MDM_G (*p* = 0.009), but no difference between KDM_G and MDM_G (*p* = 0.51). All darts were found to be empty upon retrieval. Sedation scores differed significantly between treatment groups (*p* = 0.017). The TZD_G demonstrated the highest proportion of optimal sedation scores, with 14/17 (82.4%) baboons receiving a score of 20. In the KDM_G, 12/23 (52.2%) animals achieved this score, whereas the MDM_G showed a more variable distribution, with only 3/9 (33.3%) scoring 20 (Table 1). In this group, the lower sedation scores were most frequently attributable to the following descriptors of the sedation scale [28]: “twitching fingers” for spontaneous movement, “blinking” for palpebral reflex, “decreased” jaw tone, and “only digital movements” for withdrawal reflex. In the other two groups, scores below 20 were most often associated with “blinking” and “only digital movements” for withdrawal reflex. The time to seated position and to lateral recumbency did not differ significantly between groups (*p* = 0.23, *p* = 0.49, respectively; Table 2).

The dose of propofol required for induction differed significantly between groups (TZD_G: 0.0 mg/kg, 0.0–0.0; KDM_G: 0.0 mg/kg, 0.0–1.1; MDM_G: 0.0 mg/kg, 0.0–1.0) (*p* = 0.009). In particular, the induction dose of propofol in KDM_G differed significantly from that in TZD_G (*p* = 0.007). Regarding the dose of propofol required for maintenance, no significant differences were detected between groups (*p* = 0.52). Median doses were 1.3 (0.0–2.1) mg/kg for TZD_G, 1.7 (0.8–3.6) mg/kg for KDM_G, and 0.7 (0.0–2.9) mg/kg for MDM_G. The number of propofol boluses per hour of anaesthesia was 1.1 (0.0–2.6) in TZD_G, 1.5 (0.9–2.6) in KDM_G, and 0.6 (0–2.7) in MDM_G (*p* = 0.9).

The median surgery duration, calculated only for male baboons as females did not undergo surgery, was 34 (23–35) min in TZD_G, 25 (20–29.5) in KDM_G, and 31 (23–31) min in MDM_G (*p* = 0.15). Regardless of the treatment group, no fentanyl boluses were administered during surgery. The anaesthesia duration did not significantly differ between treatments (*p* = 0.22). The TZD_G had a median duration of 118 (100–139.5) min, the KDM_G of 141 (112–161) min, and MDM_G of 104 (97.5–144.5) min. The anaesthesia duration differed significantly between males and females in TZD_G (males: 140, 137–195; females: 108, 99–116; *p* = 0.0003) and KDM_G (males: 151, 130–164; females: 112, 80–161; *p* = 0.04), but did not significantly differ in MDM_G (males: 142, 121–145; females 101, 97–108; *p* = 0.1). A significant association was found between the treatment group and the recovery score (*p* < 0.001). Baboons in KDM_G and MDM_G showed a high proportion of optimal recoveries, with 17/23 (73.9%) and 8/9 (88.9%) animals, respectively, receiving a total score of 0. In contrast, the TZD_G displayed a more heterogeneous distribution (Table 1). Recovery time varied significantly between groups (*p* < 0.001), with the longest recovery in TZD_G. The difference was statistically significant between TZD_G and both KDM_G (*p* = 0.006) and MDM_G (*p* < 0.001). Moreover, recovery time differed significantly between males and females in KDM_G (males: 11, 6–19.5 min; females: 4, 3.5–6 min; *p* = 0.004), but not in TZD_G (males: 28, 16–30 min; females: 17.5, 10.5–20.5 min) and MDM_G (males: 3, 2.5–4 min; females: 4.5, 3–5 min).

Data on arterial blood gas analysis are reported in Table 3. Arterial pH showed a significant difference between groups (*p* = 0.003), with post hoc comparisons indicating significantly higher values in KDM_G compared to both TZD_G (*p* = 0.02) and MDM_G (*p* = 0.01). The PaCO_2_ also differed significantly between groups (*p* = 0.04), with baboons in KDM_G showing significantly lower values compared to the TZD_G (*p* = 0.03). The A-a gradient and a/A% ratio differed significantly between groups (*p* < 0.001). Pairwise post hoc comparisons showed a significant difference between KDM_G and MDM_G (*p* = 0.000) for both parameters, while no significant difference was observed between ZDM_G and MDM_G, and KDM_G and ZDM_G. The HCT also differed significantly across groups (*p* = 0.03), with post hoc analysis revealing a significant difference between the KDM_G and the TZD_G (*p* = 0.036). Similarly, Hgb varied significantly across treatment groups (*p* = 0.032), with post hoc comparisons indicating a borderline significant difference between TZD_G and KDM_G (*p* = 0.056). The BE showed a highly significant group effect (*p* < 0.001). Pairwise comparisons revealed significantly higher BE values in the KDM_G compared to both TZD_G (*p* = 0.042) and MDM_G (*p* = 0.019). Lactate concentration also differed significantly among groups (*p* = 0.007). Post hoc analysis indicated significantly lower lactate levels in the TZD_G compared to MDM_G (*p* = 0.006), whereas the difference between TZD_G and KDM_G did not reach statistical significance. Creatinine levels were significantly different between groups (*p* < 0.001), with pairwise comparisons confirming significantly higher values in KDM_G compared to both TZD_G (*p* = 0.000) and MDM_G (*p* = 0.002).

**Figure 1:**
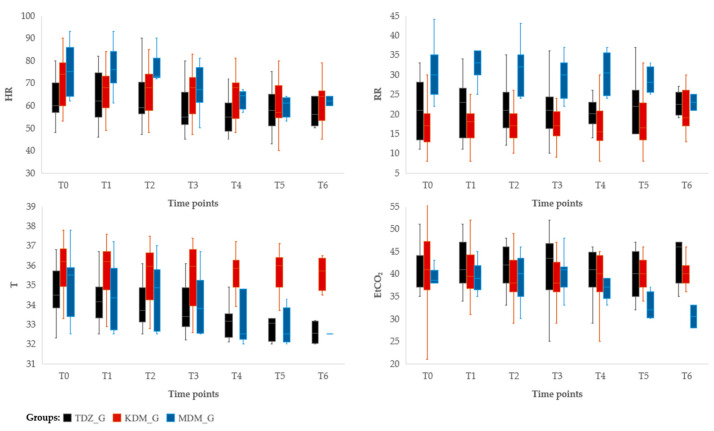
Box-and-whisker plots of the intraoperative heart rate (HR), respiratory rate (RR), rectal body temperature (T), and end-tidal carbon dioxide concentration (ETCO_2_) in 49 hamadryas baboons anaesthetized with an intramuscular combination of tiletamine/zolazepam/dexmedetomidine (TZD_G, *n* = 17), ketamine/dexmedetomidine/methadone (KDM_G, *n* = 23), or midazolam/dexmedetomidine/methadone (MDM_G, *n* = 9). T0 indicates the intubation time, and T1, T2, T3, etc., correspond to subsequent time points at 10 min intervals.

A significant effect of treatment on HR was observed (*p* = 0.001). In particular, the MDM_G exhibited significantly higher values compared to the TZD_G (B = 10.27, *p* = 0.001). Although the KDM_G showed a tendency toward higher HR values than TZD_G (B = 5.18), this difference did not reach statistical significance (*p* = 0.06). Within-group analysis revealed a significant effect of time on HR in the MDM_G (*p* = 0.001). A similar time-dependent effect was found in the KDM_G (*p* = 0.007), while no significant changes in HR over time were observed in the TZD_G (*p* = 0.21). A comparable pattern was observed for RR, for which a significant group effect was also detected (*p* < 0.001). The MDM_G showed significantly higher RR values compared to the TZD_G (B = 9.09, *p* < 0.001), whereas the KDM_G exhibited significantly lower RR values than TZD_G (B = -3.67, *p* = 0.02). In the KDM_G and MDM_G, RR decreased progressively over time (*p* = 0.001 and *p* < 0.001, respectively), while no significant time-dependent changes in RR were found in the TZD_G (*p* = 0.09) (Figure 1).

In contrast, SAP, MAP, and DAP did not significantly differ between groups. However, significant within-group time effects were detected. In the KDM_G, SAP and MAP decreased significantly over time (*p* = 0.005 and *p* = 0.012, respectively). The MDM_G exhibited a similar trend, with SAP and MAP significantly decreasing over time (*p* = 0.004 and *p* = 0.001, respectively). The TZD_G did not show significant time-dependent changes in SAP (*p* = 0.52), MAP (*p* = 0.17), and DAP (*p* = 0.24).

Finally, analysis of SpO_2_, ETCO_2_, and T revealed group and time effects. A significant difference in T was found between KDM_G and TZD_G (*p* = 0.001), with the KDM_G exhibiting a modest but statistically significant increase (B = 1.66). In the TZD_G, T significantly decreased over time (*p* < 0.001), while no significant variation was noted in the KDM_G and MDM_G (*p* = 0.23 and *p* = 0.86, respectively) (Figure 1). Regarding ETCO_2_, the MDM_G showed significantly lower values compared to the TZD_G (B = −3.00, *p* = 0.03), whereas no significant difference was observed between the KDM_G and TZD_G (*p* = 0.43). In the KDM_G and MDM_G, ETCO_2_ significantly decreased over time (*p* = 0.007 and *p* < 0.001, respectively), while no significant variation was noted in the TZD_G (*p* = 0.45) (Figure 1). No significant differences among and within groups were detected for SpO_2_ (*p* > 0.05). Differences within groups on intraoperative HR, RR, SAP, MAP, DAP, T, ETCO_2_, and SpO_2_ are summarized in Table 4.

## 4. Discussion

This study evaluated and compared the effects of three balanced anaesthetic protocols in captive *Papio hamadryas*. Consistent with the study’s hypothesis, each protocol was similarly effective in achieving a rapid and adequate sedation to permit safe approach and handling of baboons. Although some animals required supplemental darting, this did not differ significantly between groups and was most likely due to underestimation of the animal’s body weight or incomplete drug delivery associated with premature needle dislodgement within the muscle. Times to seated position and lateral recumbency did not significantly differ among the groups. However, sedation quality scores were significantly lower in the MDM_G compared to the TZD and KDM protocols. Despite the lighter level of sedation, routine procedures such as physical examination, microchip implantation, venous catheterization, and blood sampling were successfully performed, indicating that MDM protocol is adequate for minor interventions. This may be explained by the potentiation of sedation provided by methadone [35,36], whose action as a pure µ-opioid receptor agonist in the central nervous system is synergistic with the activity of dexmedetomidine on α_2B_-adrenergic receptors [37]. Moreover, midazolam might also have contributed to the overall sedative effect of the protocol due to its synergistic actions with several anaesthetic agents [20,21]. Nevertheless, the lower sedation score is likely attributable to the absence of a dissociative anaesthetic in this protocol. Indeed, in contrast to MDM_G, both the TZD and KDM protocols, which included dissociative anaesthetics, produced a higher quality of sedation, consistent with previous findings in *Papio* species and other NHPs [11,12,19]. Dissociative agents such as ketamine and tiletamine induce thalamocortical system dissociation, resulting in profound immobilization [38]. The KDM_G required significantly more propofol for induction compared to the TZD_G, which achieved the highest sedation scores and did not require propofol administration. This finding aligns with the literature indicating that ketamine, though inducing dose-dependent unconsciousness, may preserve some reflexes, thereby necessitating the use of propofol to achieve optimal conditions for orotracheal intubation [38,39]. The KDM combination resulted in deep sedation in many hamadryas baboons. Conversely, the TZD combination consistently resulted in general anesthesia, in agreement with previous studies demonstrating that tiletamine-zolazepam provides profound and prolonged general anaesthesia in NHPs, often exceeding 60 min [17]. The difference in sedation scores between TZD_G and KDM_G was not statistically significant (despite being higher on average in the TZD_G group). The fact that TZD_G achieved the highest quality of sedation without requiring any supplemental darts suggests it is the most reliable choice for effective immobilization in emergency situations, provided that its significantly slower recovery compared to KDM_G and MDM_G is taken into consideration.

In support of the study hypothesis, all three protocols maintained cardiovascular parameters within clinically acceptable limits [40], comparable to those reported in previous studies in baboons [19]. Considering that Tatoyan et al. (1972) reported a resting HR of 160–180 bpm in telemetrically monitored adults of *Papio hamadryas* [41], bradycardia was observed in all groups in the present study. This is most likely attributable to the inclusion of an α_2_-agonist in all protocols. Indeed, dexmedetomidine induces bradycardia primarily via a baroreceptor-mediated reflex in response to systemic hypertension, itself a consequence of peripheral vasoconstriction and increased vagal tone; this effect is further maintained by reduced sympathetic outflow and is typically dose-dependent [42]. Interestingly, despite receiving the highest dose of dexmedetomidine, the MDM_G exhibited significantly higher HR compared to TZD_G, and a slightly, though not significantly, higher HR than KDM_G. This likely reflects a lighter plane of anaesthesia and the shorter duration of action of the administered drugs [43] compared to tiletamine-zolazepam and ketamine [17]. The lower HR values observed in the TZD_G and KDM_G may be attributed to the myocardial depressant effects of dissociative anaesthetics [38], especially at high dosages or in combination with other anesthetic drugs, which are particularly pronounced in NHPs [17,44]. Indeed, when combined with α_2_-agonists, these depressant effects may not be adequately counterbalanced by the typical sympathomimetic properties of dissociative agents [38,45]. No significant differences in arterial blood pressures were observed between groups, although slight decreases were noted in MDM_G and KDM_G. These findings are consistent with previous studies reporting that α_2_-agonist-based protocols generally provide relatively stable hemodynamics in NHPs [11,19].

Regarding respiratory parameters, the MDM_G showed the greater variability in RR and ETCO_2_ over time, likely due to a lighter anaesthetic plan and the absence of a dissociative agent [38,39]. This group also exhibited a significantly higher RR and lower ETCO_2_ compared to the KDM_G and TZD_G. Additionally, the KDM_G showed a significantly lower RR than the TZD_G. Interestingly, despite a decrease in RR potentially indicative of clinical respiratory depression, baboons in the KDM_G demonstrated the most favorable blood gas and acid-base profile, characterized by higher pH and BE, lower PaCO_2_, a higher a/A%, and, although not statistically significant, higher PaO_2_ and SpO_2_ values. These findings suggest more efficient ventilation–perfusion matching and a more favorable acid-base balance in the KDM_G, further supporting its suitability for more invasive or prolonged procedures. Nevertheless, none of the groups exhibited signs of moderate to severe respiratory mismatching. Arterial blood gas analyses supported only mild respiratory compromise across all groups [46,47]. In the present study, ventilatory support was not required, and no serious adverse events occurred. Nevertheless, ventilatory support could become necessary in patients with apparent or previously diagnosed comorbidities, particularly when using TZD and MDM protocols. These findings are particularly notable given the inclusion of methadone in both the MDM_G and KDM_G. Although the use of opioids in NHPs remains controversial due to their potential to induce respiratory depression [7], the present results suggest that methadone, when incorporated into a balanced anaesthetic protocol for hamadryas baboons, can provide effective analgesia and enhance sedation without significantly compromising cardiopulmonary function, consistent with previous findings in other NHPs [27].

A significantly higher T was observed in the KDM_G compared to the TZD_G, which may be attributable to the inclusion of significantly heavier baboons in KDM_G. The absence of statistical significance in comparison with the MDM_G may be explained by the lower level of sedation observed in these individuals, likely associated with a higher metabolic rate, which may have contributed to the maintenance of a higher T. However, both TZD_G and MDM_G exhibited a moderate decrease in T over time, despite the use of active warming systems. A decline in T during general anaesthesia is common in NHPs [7,48], likely due to prolonged immobilization and reduced metabolic heat production [17].

Regarding blood chemistry, the significantly higher creatinine levels observed in the KDM_G likely reflect the greater number of males in this group rather than a treatment effect, as previously reported [49]; in fact, no differences were observed in BUN or urea levels. Lactate concentrations were significantly higher in the MDM_G. Although not clinically relevant, this finding may be related to the lower sedation scores in this group, which likely allowed for greater muscular tone/slight shivering and, consequently, increased peripheral perfusion. Enhanced tissue perfusion facilitates lactate clearance from muscles [50], which could partially explain the transient elevation observed. Finally, HCT and Hgb values were significantly higher in the KDM_G compared to the TZD_G (with borderline significance for Hgb). Although significant differences were observed between the two treatments, the values remained within physiological reference intervals [46,49] and were not considered clinically relevant. A drug-related effect is unlikely, as the use of ketamine or tiletamine-zolazepam in dogs has not been associated with changes in HCT values [51]. These differences may be artefactual or could reflect variations in the health status of the animals.

Consistent with the study hypothesis, the TZD_G exhibited a significantly longer recovery time, with a median duration of 19 min, compared to 6 and 4 min in the KDM_G and MDM_G, respectively. The prolonged recovery was not influenced by gender, despite males in this group having a significantly longer anaesthesia time. Furthermore, recovery quality scores were lower and more variable in the TZD_G, with several animals classified as having moderate to poor recoveries [28]. These findings are consistent with previous studies reporting that, while tiletamine-based combinations ensure effective general anaesthesia, they are often associated with prolonged and less predictable recovery phases [17,19]. In contrast, baboons in the MDM_G recovered rapidly, with 88.9% of individuals achieving the highest recovery score. These results align with those of Fiorello et al. (2009), who highlighted the benefits of a fully reversible protocol (dexmedetomidine-butorphanol-midazolam) in Asian small-clawed otters, where rapid recovery was essential for behavioral monitoring [52]. Similarly, Fagundes et al. (2022) reported faster recoveries and fewer side effects with a dexmedetomidine-butorphanol-midazolam combination in howler monkeys compared to ketamine-based regimens [21]. The MDM protocol therefore appears particularly well-suited for short procedures requiring rapid reintroduction into social groups, an important consideration in highly structured and hierarchical species such as *Papio hamadryas* [12]. Recovery in the KDM_G was slightly slower than in the MDM_G, with males showing significantly longer recovery times than females, likely due to their significantly higher anaesthesia time. Nevertheless, 73.9% of individuals achieved the highest recovery score. These findings support the suitability of the KDM protocol for procedures requiring moderate to deep sedation followed by a smooth and coordinated recovery, as previously documented for other ketamine-based protocols in NHPs [11,12].

The main limitation of this study is the lack of homogeneity among baboons within the treatment groups, including unequal group sizes. The larger sample size in the KDM_G, along with the higher proportion of male subjects, may have influenced the results related to vital parameters and recovery, possibly due to reduced heat loss associated with significantly higher body weights. Similarly, the fact that male and female subjects did not undergo the same procedures may have influenced the results, despite the absence of statistically significant differences in anaesthesia duration between groups. Another limitation is that ventilatory support could also have been guided by PaCO_2_ values, rather than relying exclusively on ETCO_2_, in order to maintain parameters within normal limits. Finally, anaesthesia was monitored by three experienced anaesthetists (>5 years in companion animals and zoo species); however, no formal inter-rater reliability test was performed prior to the study.

## 5. Conclusions

All three protocols evaluated in this study proved effective for the anaesthesia of *Papio hamadryas* undergoing routine health assessments, microchip implantation, and, in males, vasectomy. Each combination exhibited distinct characteristics in terms of sedation depth, cardiopulmonary effects, and recovery quality, reflecting the known pharmacodynamic properties of the agents used.

Among them, the KDM combination emerged as the most balanced and effective regimen, providing high-quality sedation, minimal need for propofolsupplementation, excellent cardiopulmonary stability, superior ventilation–perfusion matching, a favorable acid–base profile, and good quality of recovery in hamadryas baboons. The MDM protocol resulted in a lighter sedation and an extremely rapid and excellent recovery. These characteristics make it particularly suitable for minor or very brief procedures, especially when rapid reintegration into the social group is essential. In contrast, the TZD protocol produced a fast and deepest sedation, but was associated with prolonged and poorer recoveries. Nonetheless, due to its reliability and potent immobilizing effects, this protocol may be particularly advisable in emergency scenarios requiring rapid and safe immobilization.

## Figures and Tables

**Table 1:** Age and gender distribution (number, percentage), median and interquartile range (25th–75th) of estimated and actual body weight, and mean ± standard deviation of actual drug dosages administered in 49 hamadryas baboons anaesthetized with an intramuscular combination of tiletamine/zolazepam/dexmedetomidine (TZD_G, *n* = 17), ketamine/dexmedetomidine/methadone (KDM_G, *n* = 23), or midazolam/dexmedetomidine/methadone (MDM_G, *n* = 9).

		TZD_G	KDM_G	MDM_G
**Age**	**Juveniles (*n*)**	5 (29.4%)	5 (21.7%)	4 (44.4%)
	**Sub-adult (*n*)**	2 (11.8%)	2 (8.7%)	1 (11.2%)
	**Adult (*n*)**	10 (58.8%)	16 (69.6%)	4 (44.4%)
**Gender**	**Males (*n*)**	8 (47.1%) ^b^	18 (78.3%) ^a^	3 (33.3%) ^b^
	**Females (*n*)**	9 (52.9%) ^b^	5 (21.7%) ^a^	6 (66.7%) ^b^
**Body weight** **(kg)**	**Estimated**	10 (8.5–20) ^a,b^	20 (10–20) ^a^	10 (4–10) ^b^
	**Actual**	9.1 (6.5–18.1) ^a,b^	17.8 (8.9–21.1) ^a^	7.5 (2.9–9.5) ^b^
**Actual drug dosages** **(mg/kg)**		Tiletamine-zolazepam 3.7 ± 0.6	Ketamine 6.5 ± 1.3	Midazolam 2.4 ± 0.6
			Methadone 0.21 ± 0.04	Methadone 0.24 ± 0.06
		Dexmedetomidine 24.8 ± 6.3	Dexmedetomidine 32.2 ± 6.5	Dexmedetomidine 72.8 ± 19.5

Groups with different superscript letters (^a^, ^b^) were statistically different from each other (*p* < 0.05). In contrast, those with the same superscript letters were not statistically different from each other (*p* > 0.05). Juveniles: no signs/rudimental development of sexual characteristics; Sub-adult: partially developed sexual characteristics; Adult: fully developed sexual characteristics.

**Table 2:** Median and interquartile range (25th–75th) of time to seated position, time to lateral recumbency, time to recovery, sedation and recovery quality scores in 49 hamadryas baboons anaesthetized with an intramuscular combination of tiletamine/zolazepam/dexmedetomidine (TZD_G, *n* = 17), ketamine/dexmedetomidine/methadone (KDM_G, *n* = 23), or midazolam/dexmedetomidine/methadone (MDM_G, *n* = 9).

	TZD_G	KDM_G	MDM_G
**Time to seated** **position**	2.5 min (1.0–3.8) ^a^	4.0 min (2.8–5.0) ^a^	3.0 min (2.5–3.5) ^a^
**Time to lateral** **recumbency**	4.0 min (2.5–6.5) ^a^	5.0 min (3.0–6.0) ^a^	5.0 min (4.0–6.5) ^a^
**Sedation score**	20 (20–20) ^a^	20 (19–20) ^a^	19 (15–20) ^b^
**Recovery time**	19 min (11.5–30) ^a^	6 min (4–12) ^b^	4 (2.5–5) ^b^
**Recovery score**	9 (6–12) ^a^	0 (0–6) ^b^	0 (0–0) ^b^

Groups with different superscript letters (^a^, ^b^) were statistically different from each other (*p* < 0.05). In contrast, those with the same superscript letters were not statistically different from each other (*p* > 0.05). Sedation quality was assessed using a 20-point scale, with higher scores indicating a deeper level of sedation [28]; recovery quality was evaluated using a 15-point scale, where higher scores were indicative of poorer recovery quality [28].

**Table 3:** Median and interquartile range (25th–75th) of arterial blood gas analysis in 49 hamadryas baboons anaesthetized with an intramuscular combination of tiletamine/zolazepam/dexmedetomidine (TZD_G, *n* = 17), ketamine/dexmedetomidine/methadone (KDM_G, *n* = 23), or midazolam/dexmedetomidine/methadone (MDM_G, *n* = 9).

Parameter	TZD_G	KDM_G	MDM_G
**pH**	7.37 (7.31–7.39) ^a^	7.40 (7.35–7.43) ^b^	7.33 (7.31–7.37) ^a^
**PaO_2_ (mmHg)**	64.5 (62.9–81.5) ^a^	78.4 (63.9–88.3) ^a^	65.6 (61.6–68.9) ^a^
**PaCO_2_ (mmHg)**	55.6 (48.2–61.5) ^a^	48.4 (44.5–52.3) ^b^	49.7 (43.1–56.5) ^a,b^
**A-a gradient (mmHg)**	34.7 (21.1–47.2) ^a^	18.5 (6.7–47.2) ^b^	32.0 (26.3–52.2) ^a,b^
**a/A ratio (%)**	68.3 (57.9–76.5) ^a^	81.1 (67.5–93.6) ^b^	63.7 (49.6–73.0) ^a,b^
**HCO^3−^ (mmol/L)**	30.1 (28.6–32.8) ^a^	29.9 (27.6–31.5) ^a^	27.1 (24.1–29.8) ^a^
**Na^+^ (mmol/L)**	149.0 (147.0–150.0) ^a^	148.0 (146.0–149.0) ^a^	149.0 (147.0–151.0) ^a^
**K^+^ (mmol/L)**	2.7 (2.5–3.3) ^a^	3.1 (2.9–3.4) ^a^	3.1 (2.7–3.3) ^a^
**Ca^++^ (mmol/L)**	1.17 (1.14–1.22) ^a^	1.15 (1.08–1.25) ^a^	1.27 (1.16–1.32) ^a^
**Cl^−^ (mmol/L)**	111.0 (105.0–112.0) ^a^	107.0 (104.0–112.0) ^a^	111.5 (110.3–112.8) ^a^
**BE (mmol/L)**	3.50 (2.0–6.0) ^a^	3.90 (2.5–6.0) ^b^	0.55 (-1.58–2.75) ^a^
**HCT (%)**	38.0 (36.5–43.0) ^a^	40.0 (38.0–43.0) ^b^	38.5 (37.3–39.0) ^a,b^
Hgb (g/dL) *	13.0 (12.6–14.5)	13.5 (12.9–14.5)	13.0 (12.7–14.5)
**Glucose (mmol/L)**	7.5 (6.0–8.0) ^a^	7.0 (6.0–8.0) ^a^	8.0 (6.3–8.0) ^a^
**Lactate (mmol/L)**	0.30 (0.30–0.41) ^a^	0.51 (0.36–0.65) ^a,b^	0.65 (0.42–2.12) ^b^
**BUN (mg/dL)**	9.0 (7.5–12.0) ^a^	14.0 (6.0–17.0) ^a^	12.0 (7.5–14.3) ^a^
**Urea (mmol/L)** **Creatinine (mg/dL)**	3.3 (2.8–4.4) ^a^ 0.54 (0.40–0.75) ^a^	4.9 (2.6–6.2) ^a^ 0.95 (0.69–1.27) ^b^	4.3 (2.7–5.2) ^a^ 0.49 (0.36–0.68) ^a^

Groups with different superscript letters (^a^, ^b^) were statistically different from each other (*p* < 0.05). In contrast, those with the same superscript letters were not statistically different from each other (*p* > 0.05). * Hgb varied significantly across treatment groups (*p* = 0.032), with post hoc comparisons indicating a borderline significant difference between TZD_G and KDM_G (*p* = 0.056). PaO_2_: partial pressure of arterial oxygen; PaCO_2_: partial pressure of arterial carbon dioxide; A-a gradient: alveolar-arterial gradient; a/A ratio: alveolar-arterial ratio; HCO^3−^: bicarbonate; BE: base excess; HCT: haematocrit; Hgb: haemoglobin; BUN: blood urea nitrogen.

**Table 4:** Median ± interquartile range (25th-75th) of intraoperative heart rate (HR), respiratory rate (RR), systolic (SAP), mean (MAP), diastolic (DAP) arterial blood pressure, rectal body temperature (T), end-tidal carbon dioxide concentration (ETCO_2_), and peripheral oxygen saturation (SpO_2_) in 49 hamadryas baboons anaesthetized with an intramuscular combination of tiletamine/zolazepam/dexmedetomidine (TZD_G, *n* = 17), ketamine/dexmedetomidine/methadone (KDM_G, *n* = 23), or midazolam/dexmedetomidine/methadone (MDM_G, *n* = 9).

	Time Points	T0	T10	T20	T30	T40	T50	T60
Groups								
**HR (bpm)**								
**TZD_G**		60 (57–70) ^a^	62 (55–74.5) ^a^	59 (56.5–70.5) ^a^	55 (51.5–66) ^a^	55 (49–61) ^a^	58 (51–65) ^a^	56 (51–64) ^a^
**KDM_G**		74 (60–79) ^a^	68 (59–73) ^a,b^	68 (58–74) ^a,b^	68 (56.5–72.5) ^b^	68 (54–70) ^a,b^	57 (54.5–69) ^a,b^	64 (53.5–66.5) ^b^
**MDM_G**		75 (64–86) ^a^	76 (70–84) ^a,b^	73 (72.5–81) ^a,b^	67 (61.5–77) ^a,b^	64.5 (58.5–66) ^b^	61 (55–63.5) ^a,b^	62 (60–62) ^b^
**RR (bpm)**								
**TZD_G**		21 (13.5–28) ^a^	23 (14–26.5) ^a^	21 (16.5–25.5) ^a^	21 (16–24) ^a^	20 (17.5–23) ^a^	22 (15–26) ^a^	22.5 (20–25.5) ^a^
**KDM_G**		17 (13–20) ^a^	18 (14–20) ^a,b^	17 (14–20) ^a,b^	17 (14.5–20.5) ^a,b^	15.5 (13–21) ^a,b^	16.5 (13.5–23) ^b^	19 (17–26) ^a,b^
**MDM_G**		30 (25–35) ^a^	33 (30–36) ^a,b^	32 (24.5–35) ^a,b^	30 (24–33) ^b^	30.5 (25–35.5) ^b^	28 (25.5–32) ^b^	23 (21–23) ^a,b^
**SAP (mmHg)**								
**TZD_G**		100 (84.5–102) ^a^	93 (87–101.5) ^a^	92 (86.5–103.5) ^a^	93 (90–106.5) ^a^	91 (79–95.5) ^a^	93 (78–97) ^a^	93 (85–100) ^a^
**KDM_G**		96.5 (91–113) ^a^	99 (92–106) ^a,b^	98 (90–115) ^a,b^	96 (84–116) ^a,b^	96 (84–102) ^a,b^	98 (90.5–104.5) ^b^	105 (98.5–126) ^a,b^
**MDM_G**		111.5 (90.5–118) ^a^	96 (89–116) ^a,b^	87.5 (73–113) ^a,b^	88 (72.5–100.5) ^b^	88.5 (85–100) ^a,b^	94 (86–102) ^b^	91.5 (83–91.5) ^b^
**MAP (mmHg)**								
**TZD_G**		71 (57–79.5) ^a^	70 (65–80.5) ^a^	73 (63.5–79.5) ^a^	72 (63.5–81) ^a^	66.5 (59–76) ^a^	68 (62.5–75.5) ^a^	66.5 (59–74) ^a^
**KDM_G**		72 (65–87.5) ^a^	73 (65.5–85) ^a,b^	69 (65–80) ^a,b^	67 (60.5–82) ^a,b^	66 (62–77) ^a,b^	73 (60–80) ^a,b^	75 (65–91) ^b^
**MDM_G**		84.5 (70–92) ^a^	77 (67–86) ^a,b^	67 (59–82) ^a,b^	64 (53–74) ^a,b^	68 (60–79.5) ^a,b^	75.5 (60–87) ^b^	73 (55–73) ^b^
**DAP (mmHg)**								
**TZD_G**		60 (55.5–70) ^a^	62 (58–70) ^a^	66 (55.5–70.5) ^a^	62 (55.5–67) ^a^	60 (54–66.5) ^a^	61 (55.5–65.5) ^a^	59 (53–63.5) ^a^
**KDM_G**		61 (54–79) ^a^	66 (57–75) ^a,b^	62 (58–73) ^a,b^	59 (52.5–74) ^a,b^	57 (53–73) ^a,b^	68 (52–72.5) ^a,b^	69 (54.5–76) ^b^
**MDM_G**		75.5 (61.5–83) ^a^	70.5 (61–76.5) ^a,b^	61 (54–74) ^a,b^	58 (47–65.5) ^a,b^	63 (52.5–73) ^b^	66 (51.5–78) ^b^	66 (49–66) ^b^
**T (°C)**								
**TZD_G**		34.5 (33.9–35.7) ^a^	34.2 (33.3–34.9) ^a^	33.7 (33.1–34.9) ^a^	33.4 (32.9–34.9) ^a^	33.2 (32.4–33.6) ^a^	33.1 (32.2–33.3) ^a^	32.6 (32.1–33.2) ^a^
**KDM_G**		36.2 (35–36.8) ^a^	36.2 (34.8–36.7) ^a,b^	36 (34.3–36.6) ^a,b^	36 (34–36.8) ^a,b^	35.9 (34.9–36.3) ^a,b^	36 (34.9–36.4) ^a,b^	35.7 (34.7–36.4) ^b^
**MDM_G**		35.5 (33.4–35.9) ^a^	34.4 (32.7–35.8) ^a,b^	34.9 (32.7–35.8) ^a,b^	33.8 (32.6–35.2) ^a,b^	32.5 (32.3–34.8) ^a,b^	32.5 (32.1–33.9) ^a,b^	32.5 (32.5–32.5) ^b^
**EtCO_2_**								
**TZD_G**		44 (37–44) ^a^	41 (38–47) ^a^	42 (38–46) ^a^	43.5 (36.5–47) ^a^	41 (37–45) ^a^	40 (35–45) ^a^	46 (38–47) ^a^
**KDM_G**		41 (36.5–47) ^a^	39.5 (37–44) ^a,b^	38 (36–43) ^a,b^	38 (36–42.5) ^a,b^	40 (36–44) ^a,b^	40 (37–43) ^a,b^	40 (38–42) ^b^
**MDM_G**		38.5 (38–41) ^a^	39 (36.5–42) ^a^	40 (35–43.5) ^a,b^	41 (37–41.5) ^a,b^	37 (34.5–39) ^a,b^	32 (30–36) ^a,b^	30.5 (28–30.5) ^b^
**SpO_2_**								
**TZD_G**		93 (88.5–97.5) ^a^	96 (91.5–98) ^a^	95 (90.5–97.5) ^a^	96 (93.5–98.5) ^a^	96 (91–99) ^a^	97 (94–99.5) ^a^	93 (89–96) ^a^
**KDM_G**		95 (91–97) ^a^	96 (93–100) ^a^	96 (91–99) ^a^	95 (93–98.5) ^a^	96 (94–98) ^a^	98 (96–99) ^a^	96 (96–99) ^a^
**MDM_G**		91.5 (89–96) ^a^	96 (87–99) ^a^	97 (91–98) ^a^	96 (88.5–97.5) ^a^	94 (92.5–97) ^a^	92.5 (91–95) ^a^	91.5 (88–91.5) ^a^

In the same group, times with different superscript letters (^a^, ^b^) were statistically different from each other (*p* < 0.05). In contrast, those with the same superscript letters were not statistically different from each other (*p* > 0.05).

## Data Availability

The original contributions presented in this study are included in the article. Further inquiries can be directed to the corresponding authors.

## Abbreviations

The following abbreviations are used in this manuscript:

A-a	Alveolar-arterial gradient
a/A ratio	Alveolar-arterial ratio
ANOVA	Analysis of variance
BE	Base excess
BUN	Blood urea nitrogen
Ca^++^	Calcium ion
Cl^−^	Chloride ion
DAP	Diastolic arterial pressure
ETCO 2	End-tidal carbon dioxide
GEE	Generalized Estimating Equations
HCO^3−^	Bicarbonate
HCT	Haematocrit
Hgb	Haemoglobin
HR	Heart rate
IM	Intramuscolar
IV	Intravenous
K^+^	Potassium ion
KDM	Ketamine-dexmedetomidine-methadone combination
KDM_G	Ketamine-dexmedetomidine-methadone group
MAP	Mean arterial pressure
MDM	Midazolam-dexmedetomidine-methadone combination
MDM_G	Midazolam-dexmedetomidine-methadone group
Na^+^	Sodium ion
NHPs	Non-human primates
PaCo_2_	Partial pressure of arterial carbon dioxide
PaO_2_	Partial pressure of arterial oxygen
pH	Potential of hydrogen
RR	Respiratory rate
SAP	Systolic arterial pressure
SC	Subcutaneous
SpO_2_	Peripheral oxygen saturation
T	Rectal body temperature
T0	Intubation time
TZD	Tiletamine-zolazepam-dexmedetomidine combination
TZD_G	Tiletamine-zolazepam-dexmedetomidine group
